# Making my voice and owning its future

**DOI:** 10.1136/medhum-2024-013021

**Published:** 2024-11-11

**Authors:** Jamie Preece, Emma Sullivan, Fin Tams-Gray, Graham Pullin

**Affiliations:** 1 Barnsley Assistive Technology Team, Barnsley, UK; 2 Unaffiliated, Barnsley, UK; 3 Duncan of Jordanstone College of Art and Design, University of Dundee, Dundee, UK

**Keywords:** Medical humanities, disability, design, Digital Technology

## Abstract

This article explores disabled experience and the future of technologies relating to augmentative and alternative communication (AAC). This field includes people’s use of AAC devices, typically in combination with other modes of communication, including vocalising, revoicing and body language. Such devices have speech technology and digital voices built into them and we will consider who could be said to have ownership of these technologies. We will also explore the role that people who use AAC have in making their AAC—and how this also contributes to shaping its future. The meanings of ‘voice’, ‘making’ and ‘ownership’ in the context of AAC are many. Yet too often the relationship between these is presented as if it is singular and straightforward. This paper will start by considering the most prevalent, obvious interpretations and build alternative and more complex directions from there. One of the authors uses AAC and is constantly personalising his software, editing and remaking it to reflect his needs and current thinking, representing his voice in ways that he feels ownership of; another is a life partner and can also be thought of as being part of his AAC. Two authors are researchers in an art school, where the act of making things in studios and workshops is inseparable from creative authorship and ownership. Together, all four authors are exploring the meaning and making of speech technology, experimenting with and appropriating it in ways not anticipated by its developers. This paper is a hybrid of voices: disabled and non-disabled; academic and non-academic coresearchers; designers and codesigners. Its unconventional format is intended to reflect the unconventional relationship between the researchers and to represent the conversation between these different voices.

## Introductions

 Augmentative and alternative communication (AAC) includes—but is not defined by and is not limited to—the use of speech technology devices by people who do not speak. The first author of this paper is a person who uses AAC. Before further introducing AAC, we should say a little about how we worked and wrote together as four coresearchers because this will explain the slightly unconventional form of this paper and the meaning of its typographic treatment.

The writing of this paper was collaborative, yet unconventionally so: the first author uses AAC and does not read; two of the authors have experience of supporting a person using AAC; the other author has no lived experience of communicating through AAC. We evolved two different ways of writing together. The paragraphs in bold text throughout this paper were initiated by Preece and Sullivan drawing on their lived experience of disability and of using AAC. Combining sentences previously composed by Preece with utterances made during our discussions, we edited these together into sections of roughly a hundred words each (Berlant and Stewart 2019). This was short enough to be crafted word by word with Preece—we read them together and rewrote them until Preece was satisfied—yet long enough to balance a necessarily longer research article. The remaining body text was drafted by Tams-Gray and Pullin as design researchers and then read out, reviewed and edited by us all. The sections in bold are written in the first person singular, whereas the rest of the paper is typically in the third person (or first person plural when describing our research activities and writing), so this too will signal the different perspective. The evolution of the paper has also been a full collaboration, with all four authors participating in the online workshop and in-person symposium that were part of the path to publication process for this issue.

AAC encompasses a range of ways of supporting interpersonal communication and social interaction. This can combine spoken or other forms of communication and involve gestural or symbolic languages, and physical and digital tools and devices (Alper 2017). This paper will mainly focus on ‘speech-generating devices’, also known as ‘voice output communication aids’, which rely on speech technology, including text-to-speech synthesis. For the sake of consistency—and to avoid conflating AAC and speech technology—we will use the term ‘AAC device’ for AAC using speech technology. So when Preece refers to ‘my AAC device’ in the sections in bold text, he means his speech technology device. In this paper, a ‘digital voice’ will be used to mean a particular-sounding text-to-speech voice that can speak any text that is typed, composed or imported, its particular sound including how words and phrases are pronounced and articulated and also the auditory qualities of the voice—all of which we will return to later.

**My partner, Emma, is a part of my AAC. If Fin and I were to go into a meeting, neither of us would be able to plan exactly what we were going to say. However, as an AAC user, sometimes this is expected of me: that I pre-prepare every utterance. Emma re-voicing for me allows me to share ideas on the spot; there may be times when a knowing look in her direction says all that’s needed to be said, faster and easier than using my digital AAC device. It’s just part of it, it shouldn't be separate. It’s all part of the whole thing.**

As part of AAC, speech technology devices will typically be used (if used) in combination with vocalisation, gesture, body language and facial expressions, with all of these modalities contributing to the interpersonal interaction (Higginbotham 2010). Indeed, in a paper entitled ‘A competent speaker who can’t speak’, the linguist Charles Goodwin described how Rob, a man with aphasia who had almost completely lost the ability to speak, used not only his body language and facial expressions but also ‘the language resources provided by the speech of others’ to communicate and to participate in social interactions (Goodwin 2004), which suggests that AAC should not always replace, but rather work with, these other modalities.

Among people who do use AAC devices, some will compose sentences using a keyboard of some kind; others compose or select utterances using symbols or menus. Audible speech is then generated by the speech technology. One perennial issue in AAC is the length of time that it takes to compose an utterance and how this can exclude someone using AAC from the flow of a conversation (Higginbotham, Fulcher, and Seale 2016). This is another issue that we will return to later in this paper.

**Figure 1:**
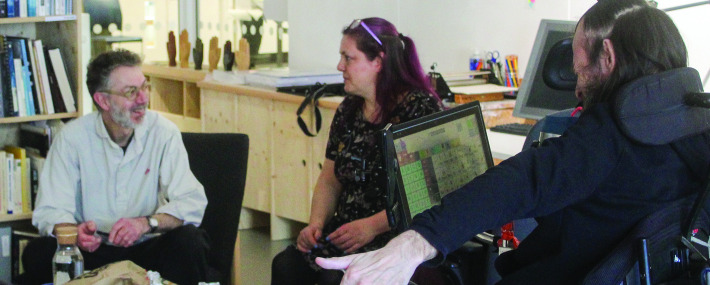
From right to left Jamie Preece, Emma Sullivan and Graham Pullin sitting in Studio Ordinary in Duncan of Jordanstone College of Art and Design. Alt Text: Three people are seated in conversation: a man in a motorised wheelchair with an AAC device attached, a woman with purple hair and another man wearing glasses. They are surrounded by art materials and books, in a studio with a creative and convivial atmosphere. Consent or permission: The image shows three authors of this paper (the only people shown—and it was taken by the other author) who hereby give permission for it to be reproduced in this paper.

Our research methodology was based on design research methods and will be described in more detail later. Yet more than anything our collaboration was framed by another AAC user’s advocacy of ‘mentorship’: that researchers should not only involve but *spend time* with people who use AAC, creating the opportunity for mutual learning and teaching (Portnuff 2006). This transcended even the time that we were actively focused on conceiving, making (Volkmer and Broomfield 2022) and reflecting on various forms of prototype together (Figure 1). This paper revolves around Preece’s mentorship, although the project as a whole has also involved other AAC users as mentors.

## Making my AAC my own

Current AAC devices typically run on digital devices, accessed by touch, keyboard, switches, head tracking or eye tracking. Most on-screen user interfaces are based on grids of words or phrases or images, built up into different pages of options. This modularity means that they usually can be edited, but Preece has gone further: rebuilding his entire interface.

**I was told to edit rather than create from scratch, but it didn’t work for me, I wanted things where I wanted them. Looking back now, I feel if I hadn’t done it my way I might have given up and not become an AAC user. The more I collected and organised my own words, the more I found I could organise my own thoughts. There were many words I knew but had never been able to use as I could not vocalise them. I needed to put them in an order that made sense to me.**

Preece himself uses an illustration of library shelves to convey this (Preece and Lee 2023) and creating one’s own AAC is like organising a personal, private library. The organisation may also reflect not just needs but also interests, priorities and personality. As an instance of this, Preece has several pages of his communication interface—several bookshelves—devoted to swearing.

Being able to swear when I want and how I want is important to me. For36yearsI relied on people interpreting my speech and gestures, often changing my words in the process. I would say“He’s a f***ing bastard” **but the person revoicing me might censor this and say “Jamie doesn’t like him**” **instead. Which is not the same thing. So I take swearing quite seriously and have created several pages of different ways to swear. The details matter! Although some people hate it, I like the way that I can use swearing to add emotion or humour to my communication.**

Swearing is both a perennial issue and still an overlooked area in AAC, something that AAC has had an awkward relationship with (Alm and Ellis 1999). Historically, text-to-speech devices have even censored swearing, speaking euphemisms in place of typed expletives (much as the automatic transcription of online meetings censors what is said—as we found out in the online conversations that were part of our coauthoring process on this paper). The censoring that Preece highlights is part of a complex relationship between coconstruction, collaboration and individual agency (Kurlenkova 2021).

**I have a whole page for the different ways I might want to ask for a cup of tea.“Will you make me a cup of tea please?”;“Any more tea in the pot?”;“Any more tea in the pot darling?”;“Can I have more tea please my love?”;“Did you have a look if there was any more tea for me?”and so on—that’s only 5 out of 21! I also like having lots of different ways to say good morning to people at work. Do you say it exactly the same every time?**

Especially in the context of a neutral read style of synthesised speech, a variety of ways of saying something is needed to convey spontaneity and thoughtfulness.

Communication devices have for a long time been framed within a sender–receiver conceptual model, as a simple text-to-speech stage of a chain of communication. There is increasing appreciation that this is part of multimodal interaction (Higginbotham 2010), just as Preece’s communication includes his own voice, Sullivan’s voice, his body language as well as his AAC. AAC is as much a medium as a transmitter: it helps to frame and shape our thoughts as well as convey them to others.

**It is more than just a voice—it’s the voice of my thoughts and my identity. I now think more in words. That may sound strange but I didn’t used to. Words are more than just talking tools, they are thinking tools. I didn’t used to have an inner voice. I had ideas floating around but without the words attached to them. I now have an inner dialogue and have the words I need to talk through situations in my head as well as out loud. I now dream in my communication aid voice. It was the start of a new life for me, a new way of existing.**

## Choosing and identifying with a voice

**I use a digital voice called‘Graham’which suits me, but lots of other people—including many of my AAC user friends—have the same voice. This makes it feel less my own. If you think about it, we all say things in our own unique individual way. It can also be confusing. For instance, imagine you’re in a meeting and come up with a great idea and someone else gets the credit simply by virtue of having the same digital voice. However, this can prove to be quite amusing at AAC events.**

Colin Portnuff was an engineer who lost his speech through motor neuron disease (MND), a very different life experience to Preece’s, who lives with cerebral palsy. Portnuff used AAC with—at the time, it being 2006—a generic American male adult voice. In a deep and illuminating talk to researchers in the USA, he noted that ‘I guess I am beginning to identify with the voice myself’ (Portnuff 2006). ‘Beginning’ implies a relationship developing over time, through familiarity and by association.

Portnuff reflected ‘It is only natural to associate voice with identity, but I think the professionals doing, and guiding, research should be cautious about the flip side. Do you really hear the individuality of each speaker who uses the same voice?’

Another person who became associated with a computerised voice was of course the physicist Stephen Hawking, the most internationally famous person to have used AAC. Hawking could be said to have felt (and exercised—we will come back to this) ownership of his voice, even though it had an American accent, whereas he was British, and by today’s standards was obviously artificial sounding. Perhaps, given his field of cosmology and the era of the Space Race, this appropriately evoked crackly countdowns broadcast from Cape Canaveral? Either way, and in the context of his fame, it became his trademark and part of his identity. As a result, however, other people using a similar voice were correspondingly denied ownership in turn. ‘It was like walking into a room full of Stephen Hawkings’ as another AAC user described a conference reception.

**When I first started using an AAC device, I had to choose a voice. Lots of options come pre-installed on most AAC devices, and I chose a voice called‘Graham’. I chose it because it was half alright. It is clear and intelligible, which was the main appeal for me, and British, not American. It was the best of the choices available to me—the least unsuitable. Although now,12yearson, I wouldn’t change it. Even though I didn’t realise until many years later that so many people have chosen the same voice. Because to me, it is now my voice.**

For most people using AAC devices, the choice is between existing voices made by companies including Acapela, Cereproc and SpeakUnique. These voices are created by recording a voice actor reading from a script—usually speaking in a (supposedly ‘neutral’) reading style—from which a database is compiled. From this, a digital voice is created that can be installed and used within a text-to-speech and speech synthesis engine, and thereby within different applications, from screen-readers to AAC apps.

As text-to-speech has evolved, so have the priorities for its development. Originally, basic intelligibility was prioritised; once this was reasonable, the emphasis became to be ‘realistically’ human sounding, rather than robotic, realised through more sophisticated sampling of larger databases of recorded speech.

Subsequently gendered male and female voices were developed; child as well adult voices; voices from different countries; voices from different cultures or subcultures. In 2008, Cereproc released Heather, a Scottish (rather than an English or American) female voice with Stuart, the world’s first Scottish male voice, following in 2011; in the same year, Acapela introduced Saul, described by them as ‘US English—“hiphop”’. This evidences a well-recognised need for a diversity of digital voices, so that people using speech-generating devices might choose a voice that they feel represents them. New voices are being proliferated, with different regional, cultural and gendered qualities and identities.

**I am a proud Yorkshireman: it’s one of the first things I tell people about myself; it’s the first thing I wrote in my bio for this paper. Being a Yorkshireman is an important part of my identity. A digital voice with a Yorkshire accent has been created in the last couple of years, that people assume that I would want to adopt. I have tried it out—but it’s from the wrong part of Yorkshire. Yorkshire is a big place: if you meet someone from Barnsley and someone from Sheffield the accents are so different. So it is not a voice that I can identify with. I am not that Yorkshireman.**

One issue comes from just how nuanced our perceptions of voice can be. It seems that there are so many ways that a voice can be ‘wrong’ to someone. In ways reminiscent of Masahiro Mori’s ‘Uncanny Valley’, the closer it gets to an obvious ‘match’, the more jarring it seems that these dissonances can be (Mori 1970).

## Restoring a voice

In mainstream as well as assistive technology, there is the concept of ‘voice banking’: recording samples of someone’s speech, so that a synthetic digital voice might be generated from this. At the time of writing, prominent examples of this include Apple’s ‘Personal Voice’, a voice-banking service built into their newest operating system, which allows iPhone users to preserve and digitise their voice by reading a 15 minute text prompt (Apple 2024).

Voice banking is already part of AAC too: in 2010, Scottish speech technology company Cereproc was able to create a voice for American film critic Roger Ebert following his loss of his speech from throat cancer. What made this a special case was that, due to his profession, there were hours of broadcast-quality recordings of Ebert speaking available to the speech technologists. This was at a time when it would not have been obvious to create a voice archive—and had already been created by the time that Ebert’s voice was affected by his illness. Ebert died in 2013, but his reflections on his computerised voice are still available (Ebert 2010). What is striking—and chimes with Preece’s reflections on the ‘Yorkshire’ voice—is how important the nuances are: one of the things that Ebert appreciated was that ‘For one thing it knew exactly how I said “I”’.

SpeakUnique, a University of Edinburgh spin-out company, offers a service to people in the UK. Developed with the MND association. “I Will Always Be Me” is an e-book that describes what a diagnosis of MND might entail for someone (SpeakUnique 2022). By reading aloud the story, the user is also able to bank their voice in less than half an hour. The recording can also be used to create a digital version of the individual’s voice that could be installed in a speech-generating device that they might use in the future.

While the potential for voice banking is now ubiquitous, it is not as accessible to those who have never had a speaking voice as well as those who have already lost one that they might have had. In contrast, VocalID pioneered a service offering customers the ability to create a bespoke voice that can imbue a voice chosen from a crowdsourced collection with some of the qualities of any vocalisation from the eventual user of that voice (VocalID 2024). Founder Rupal Patel recalls witnessing a little girl and a grown man having a conversation using their AAC devices: these were different devices, but with the same digital voice. ‘We wouldn’t dream of fitting a little girl with the prosthetic limb of a grown man, why then was it okay to give her the same prosthetic voice as a grown man?’ (VocalID 2024).

Interestingly, this is an example that challenges the so-called ‘trickle-down effect’ whereby mass market innovations eventually are expected to benefit disabled people (Pullin 2022). VocalID was initially conceived in the context of AAC, for many of the reasons already discussed, yet is now finding wider commercial applications in representing brands—*brand identities*—through carefully crafted voices.

Voice banking is another technology that is dominating how people think about AAC: indeed Preece has been asked whether his digital voice is ‘his own voice’, synthesised. Yet at the same time, is there something of a medical model of disability behind such a literal framing of the ownership of an AAC voice? We question an implication that any synthesised voice can, or necessarily should be, an exact—an inevitable—replica of their voice. Especially if the person using AAC has never spoken in that voice.

## Tones of voice

The emphasis on ‘voices’ also obscures another perspective: that the qualities that define our voices vary not just from individual to individual but also from context to context—we adopt different tones of voice in different company, in different situations and in different moods—and from utterance to utterance. The very text-to-speech technology at the heart of speech-generating devices is not a neutral medium: it inherently privileges the words said over how they are said. The person using the technology only gets to specify the words—perhaps with the addition of punctuation such as a question mark or exclamation mark.

When Colin Portnuff said ‘I guess I am beginning to identify with the voice myself’, he went on ‘but I would still not hesitate to toss out the voice I use if I could get a more expressive one’. Portnuff spoke of his wish ‘to be able to sound sensitive or arrogant, assertive or humble, angry or happy, sarcastic or sincere, matter of fact or suggestive and sexy’ (Portnuff 2006).

Lots of research are focused on expressing emotions (like happiness, sadness, anger and fear—so-called primary emotions) through speech synthesis, as if this encompasses all that is conveyed with tone of voice. But Portnuff’s tones are more diverse and more nuanced than this: only angry and happy are simply emotions; sensitive and suggestive—indeed most of the qualities he mentions—are more contextual and socially constructed. They involve and imply the conversational partner as well as the person talking.

It was by involving his conversational partners and coconstructing meaning with them that the man with aphasia introduced earlier was a competent speaker. Despite a limited vocabulary of three words, he was able to intone these words in a nuanced way, for example, using the word ‘yes’ ‘as a textured, non-binary answer’ with which he could steer a conversation (Goodwin 1995). The meaning of this intonation—and its conversational consequences—was coconstructed.

The phonetician John Laver explored how the nuances of the qualities of a voice can be described, by experts and by laypeople (Laver 1974)—an endeavour that continues to this day (Garellek 2018). His *labels for voices* contain both objective definitions and inherently subjective labels. Objective phonetic definitions include ‘modal’ and ‘creaky’ voice which, while usefully universal, are obscure to anyone but phoneticians or speech scientists. Laver refers to adjectives such as velvety, rich, golden, flat, thin, bird-like and thick as ‘wholly impressionistic labels’—yet accessible to laypeople. Another distinction that Laver makes is that whereas phonetic labels deliberately and precisely isolate a single quality of speech, impressionistic labels tend to refer at once to several features. Yet it is these that feel the most interesting in the context of AAC because they are nuanced and accessible.

This reminds us of other qualities that resist objective definitions. The musician Brian Eno wrote about his fascination with perfume and his attempts to discover—or draw—a map that related scents to each other. ‘Where does karanal stand in relation to tuberose? Or sandalwood to sage? (Eno 1992)’. ‘Don't ask me’ he eventually conceded. Yet his realisation that this probably wasn’t even possible was itself a creative beginning that he took back into his music.

We are minded to bring it into AAC. If speech technologists tried engineering voices which might defy definition, except subjectively, then impressionistic labels would be needed instead. If these were created and applied by people using AAC then, rather than being a failing, this could open up new models of research and ownership (Pullin 2015).

In our design research, we are playing with how we might visualise these complex and elusive qualities of tone of voice, drawing on the practice of information design (Tufte 1990). Our hope is that this could have several roles: to engage people in more nuanced conversations about tone of voice; to act as icons on a communication device to help people select tones of voice quickly in the midst of a conversation. A third role, to underpin a computer application to let people craft their own tones of voice, we plan to return to in future research.

## Using different voices

According to the concept of speech acts (Austin 1962), our speech is not just used to convey information but also to ‘do things’: to greet, to swear (in both senses: to promise or to curse), to perform. Here tone of voice can be even more important.

**If I’m being romantic, I don’t want to sound robotic. I want to show Emma my love for her. If I’m angry with someone, swearing in my usual voice sounds too polite and people might think I am joking. If someone has died I want to be able to say“I am sorry”and sound like I mean it, but I know that my digital voice sounds too matter-of-fact and I would be worried that I would come across as being insensitive rather than empathetic.**

Returning to swearing, there is an irony that although Preece now has the freedom to speak any word he wishes, his text to speech technology undermines him in a different, less expected way: the ‘neutral’ delivery of his computerised voice can make swearing sound ironic, insincere, humorous even, which compromises its role as a speech act (Austin 1962).

Would you say“Do you want a cup of tea?”in the same tone of voice as you want to say“Do you want to come to bed?”Or should“F*** off”and“F*** me”sound the same? We need to start a conversation that is currently not happening enough, if at all. A conversation about the identity, sexuality and individuality of people who use AAC and about how AAC could do more to support this aspect of their lives. People need AAC to give them the freedom to create and explore their sexuality.

We are interested in the concept of code-switching, a social strategy recognised by minority cultures in which they might adopt a more mainstream accent or way of speaking in the company of a majority; in other words, deliberately toning down their cultural identity in circumstances in which this might risk marginalising them (The Guardian 2024). Just thinking about this possibility makes it clear that the voice, like communication itself, does not only concern an individual in isolation.

Incidentally—and confusingly—when code-switching is referred to in AAC research, it denotes something different: the particular case of multilingual AAC in which people are switching between different languages, and in connection with this between different digital voices each with their own language model, vocabularies and pronunciation. It is this that is termed ‘code-switching’ in the AAC literature (King and Soto 2022). The authors of that paper use the term code-switching, ‘recognising that differences in terminology can be meaningful from different theoretical perspectives’. Perhaps it is also telling that the field of AAC chooses a linguistic rather than a social perspective from which to define that terminology? Perhaps this indicates the field’s priorities and how little attention has historically been given to the nuances of social interaction?

## Conceiving new voices

The idea of changing one’s voice in different circumstances is one that is being explored by speech technologist Éva Székely. Starting with voices created from recordings of formal ‘read’ speech and more informal ‘conversational’ speaking style, she is developing ways to allow blends to be made between these. In this way, people who use AAC might have control of more nuanced tone of voice. The ability to create different voices by combining two different (more generic) synthetic voices could provide a way of taking ownership of digital voices that were not themselves individually created. For example, Székely and other speech technologists are already blending male and female voices to represent non-binary and gender-ambiguous personas and identities (Székely, Gustafson, and Torre 2023).

Having the agency to select the voices and decide on the *reason* for blending them could be profound, as borne out by conversations with several of our other mentors: one young person we have spoken with would like a new synthetic voice, but her family are resistant to this change. Perhaps—openly or secretly—she might gradually phase her new voice in so that her friends have a chance to get used to it (or even so that they might not notice the transition?). Code-switching that has been considered in AAC in terms of moving between different languages could also apply to more nuanced cultural variations within the same language, in different company. In this way, people could meaningfully make their own voice, on their own terms.

We find ourselves in a situation in which speech technology lags our imagination. Parametric control of speech synthesis (through markup languages such as VoiceXML or SSML: ‘speech synthesis markup language’) has been theoretically defined for decades. Yet the voices used in AAC still afford control of only baseline pitch and word rate. Much of this is because the technology has been developed for mainstream text-to-speech applications in which the words are the only ‘information’ available. If an entire book or website or database is available as a text file, there will not be any supplementary data on how it might be spoken, which will need to be inferred from the text itself. AAC is very different. In the first case, speech is a means to accessing written text; in the second, text is a means to an end of realising speech. We see fascinating parallels with fundamental concepts of *logocentrism* and *phonocentrism* that we are beginning to explore, but which there is not the space in this paper to do justice to (Derrida 1976).

In AAC, not only the words are available but also their author. This individual may know how they want to sound and—given how important this is in conveying their social presence and conversational intent—be prepared to invest the additional attention needed to direct this. We have started to imagine hybrid digital voices and digital-human voices that could open up more complex control. In both cases, the unique context of AAC, together with the perspective and investment of its users, mean that new directions might emerge that in turn influence speech technology as a whole.

## Performing with voices

**“Hello hello hello. Hello everyone. How are you this evening? Come on, is that the best you can manage? Are you enjoying your evening? It’s great to be back here in the Communication Matters conference. I have to admit I'm nervous. Can you tell from my voice? I haven't done a comedy gig for ages. So please be kind to me. If any AAC users want to heckle just send me a text and I'll have a great comeback ready by tomorrow night. But one advantage I have with using the device is that I don't have to worry about fluffing my lines.”**

Preece also does stand-up comedy. A lot—but by no means all—of his material derives from his disability and his use of AAC. When he asks the audience whether they can hear his nervousness, this is doubly ironic: since his digital voice is not expressive, and also because Preece is a confident and consummate performer in the first place.

At the moment he carefully composes and crafts his act and stores it in his AAC device. On stage he has a foot pedal, each press of which speaks the next utterance, according to how he has partitioned his script, but no further. Another conscious pedal press is needed to advance to the next. In this way, he can pace his act: waiting for audience laughter to die down before giving his next line; sometimes even splitting up a sentence to hold back a punchline for dramatic effect. Always reading the room and having the audience eating out of his hand.

As you might imagine from the previous section, Preece employs a fair amount of swearing in his comedy routine, as he does in his everyday life. Again, there are times when the juxtaposition—the contradiction—of bad language in a relentlessly polite voice is itself amusing. Yet there are other times when the swearing is more pointed, more angry—and would land differently were it delivered with more aggression. Or more quietly and even more deliberately; either way, delivered differently.

“You know, sometimes people feel sorry for me. They pat me on the head and say they wouldn't be able to cope if they were like me. The twats. What do they know? I may struggle with some things, but I have a privileged life. Let me tell you about my privilege. I know I'm treated better than some, I'm very aware of that. It’s been going on all my life, I'm pretty used to it. So f*** your sympathy I don't need it. I've got spaz privilege. Yes, spaz privilege.”

**Figure 2:**
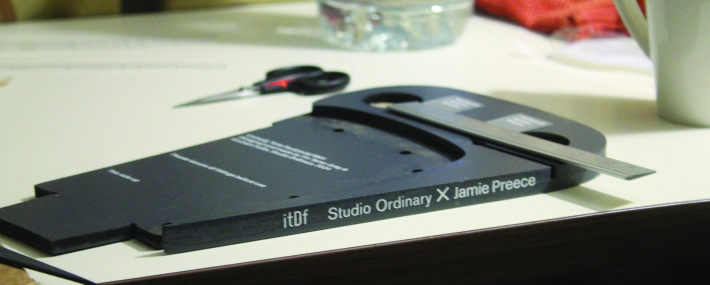
Work-in-progress on Jamie Preece’s custom foot rest in Studio Ordinary. Alt Text: A charcoal grey foot plate is being assembled on a workbench. It has a recess for a wheelchair foot rest and two more for round switches. Instructional text in white has been applied to its surfaces. Around the edge it is branded 'itDf' and 'Studio Ordinary X Jamie Preece'.

We have all discussed this and Preece would value more control over his line-by-line delivery. So one of our experiments together is a foot rest with two foot switches for Preece’s comedy (Figure 2). One switch is used as before to choreograph the act; the second switch might allow Preece to change his voice, his delivery, either to suit the material or to respond to the reaction of his audience.

**Figure 3:**
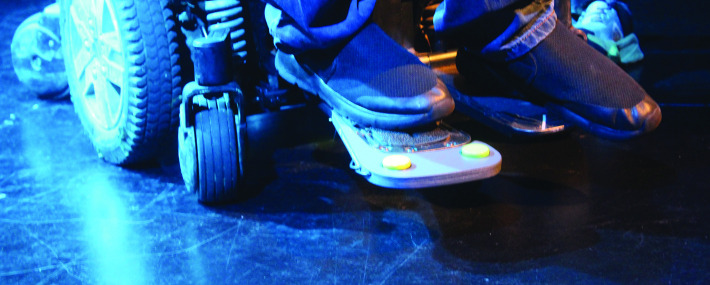
Jamie Preece using his custom foot rest in his comedy act. Alt Text: Under blue stage lighting that floods a scratched black floor, Jamie Preece is in the middle of his act. We see his feet only: resting on the foot plate of his motorised wheelchair, his right foot is within reach of two round switches, one yellow and one green. Consent or permission: The image shows (the feet of) one of the authors of this paper, who hereby gives permission for it to be reproduced in this paper.

**I would want my new custom-made foot rest and foot switches to be nice and light and functional. And not to be annoying. It would be reliable and help me avoid technical difficulties during my standup routines by prioritising functionality. Currently, my foot rest barely has enough room for my foot and the switch, so I need more space. Ideally, it’d be longer rather than wider, and would sit on the right-hand foot rest because that’s the foot I use to activate foot switches.**

Our approach has been to realise a platform that Preece can experiment with and take ownership of. Perhaps its eventual use will be unforeseen by any of us. Initially it was used to address an issue he experienced where he would accidentally press the foot switch two times, which would result in a line being missed from the comedy routine—sometimes skipping straight to the punchline of a joke without the set up. During an initial prototype fitting session, Preece programmed the second foot switch to amend this by halting the read speech, returning to the previous line and resuming from there. After roughly a month of owning the prototype and experimenting with it, including using it in public performances (Figure 3), Preece reprogrammed the switches to allow him to intone his voice as well, reading lines in a deeper and slower voice than usual, which has even more effect on the audience for being so unexpected. This gives him even more agency—and, therefore, accountability for his language.

## Alternative futures for AAC

When AAC receives attention in the press, it is often because of a story about a technological breakthrough. ‘Scientists create decoder to turn brain activity into speech’ ran the headline on a story about surgical experiments being carried out in San Francisco (The Guardian 2019). Referencing brain-computer interfaces (BCIs) in the form of brain implants, the article predicted ‘speech at the speed of thought’ in the future. As a result, many people assume that this is the future of AAC.

Yet this assumption risks reverting to a medical model of disability in which the implicit role of technology is to fix disabled people rather than to reflect disabled experience. What Ashley Shew has labelled ‘technoableism’: an implicit responsibility on disabled people to ‘fix’ themselves by any means available (Shew 2023).

But who gets to define these futures? (Charlton 1998). The aforementioned article quoted the late French journalist Jean-Dominique Bauby, who wrote his memoir using AAC, but no living person using AAC was interviewed alongside the scientists and surgeons. Disabled people are framed as medical subjects in the research, as patients of future treatments and recipients of future technologies. This research will of course continue, given its scientific insights and applications. But how can we imagine alternative futures?

## Envisioning the future

**Figure 4:**
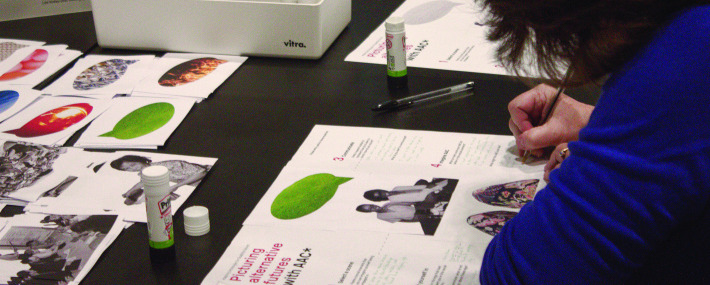
Creating a collage for the 'Picturing alternative futures with AAC' activity. Alt Text: An adult is absorbed in thoughtfully annotating a piece of paper onto which they have already stuck cutouts of a grassy speech bubble, two seated figures and the legs of a pair of floral trousers. Glue sticks, pens and other paper cut-outs are scattered around the table top. Consent or permission: The person in the image is not identifiable—and gave permission for the photograph to be taken.

We have tried picturing alternative futures with AAC using collage: providing cutouts of different people, locations and speech bubbles with which participants can compose future scenarios and so explore the role that their AAC might have in these (Figure 4). Collage allows ideas to be visualised, yet without needing to draw, which some people do not do or do not feel comfortable doing. This is aligned to exploratory research methods such as ‘cultural probes’ (Gaver *et al*. 2004), which are now beginning to be employed in AAC research (Ibrahim *et al*. 2024).

We also use prototyping as an exploratory method. In an analogous way that AAC is not just a communication tool but also a thinking tool, so too are prototypes not just tools for testing but tools for speculation and reflection. Different types of prototypes can either allow us to immerse ourselves in an experience or to step back and be more conscious of the assumptions and values that might be built into technologies that we might otherwise leave unquestioned.

One of these methods is ‘experience prototyping’ in which designers and the people they are designing with try out new experiences without realising them fully (Buchenau and Suri 2000). Experience prototyping can save time or money to create a fully working prototype but (most interestingly) can also be used to explore speculative technologies or situations. It can be done in different ways: typically taking advantage of accessible and low-cost computing modules (like Arduino or Raspberry Pi) and hacked or scripted interactions; sometimes even resorting to ‘Wizard of Oz’ prototyping employing hidden human intervention. One example of the imaginative use of experience prototypes in AAC is the *Magical Musical Mat,* which translates interpersonal touch into sounds. This was designed with autistic children and their families, developed through iterative prototyping (Chen 2024).

AAC relies on technologies that are primarily focused on other markets. Although this means that they are not being consciously shaped with AAC in mind—as a field, let alone at an individual level. Text-to-speech itself is primarily being developed for entertainment, internet content and digital voice assistants. Some capabilities that have been defined for decades have never been made available, perhaps because their value to these key applications is unclear. More recently—or not so recently (Waller 2024)—artificial intelligence (AI) has the potential to revolutionise AAC alongside all kinds of domains, yet is an industry of its own; it can seem as though its future direction is beyond the influence of a field like AAC. It is developing at such a pace that developers in other fields may struggle to integrate it with the technologies of their own. There is an intense focus on AI in AAC and some of the controversies around its use are beginning to be explored and debated (Valencia *et al*. 2023).

Experience prototyping could allow design teams to play with relevant technologies, exploring possibilities and sharing speculative concepts with users to inform how those technologies are shaped. Prototypes can be influential ‘documents’ of design intent, embodying design visions within technical cultures (Buchenau and Suri 2000). Allowing people to immerse themselves in an experience can illuminate issues that are not the expected goals of technological ‘progress’ and already on the agendas of technology developers.

## Critiquing the future

The role of designing and prototyping can be indirect and at the same time critical.

**One time I was messing about with the settings on my AAC device, and changed the sound of the voice. I realised that I could make it sound like a person with cerebral palsy. It sounded like I might talk, if my voice was clearer. Part of my identity is being a disabled man. This is not something that I can or want to hide. I am proud of who I am. My friends and family love me for who I am. No one is talking about whether this could be part of my digital voice.**

Here, a prototype voice made by Preece has embodied an issue—in this case, disabled identity—in a thought-provoking way and catalysed a discussion about something that is not being discussed enough.

In our research together, we have discussed many issues around AAC. Some of these are rather elusive and resist discussion or can be difficult to keep the conversation on, once started. These include intangible issues such as timing, turn-taking and waiting (Higginbotham and Wilkins 1999; Higginbotham, Fulcher, and Seale 2016). It can be easy to always focus on trying to make AAC faster, not confronting the reality that it produces different rhythms of interaction than non-augmented communication. Another part of our practice has been to embody just these issues in physical—and therefore tangible—prototypes. As a method, this has a lot in common with ‘critical design’ defined by Tony Dunne and Fiona Raby as design that asks questions rather than provides solutions (Dunne and Raby 2001).

**Figure 5:**
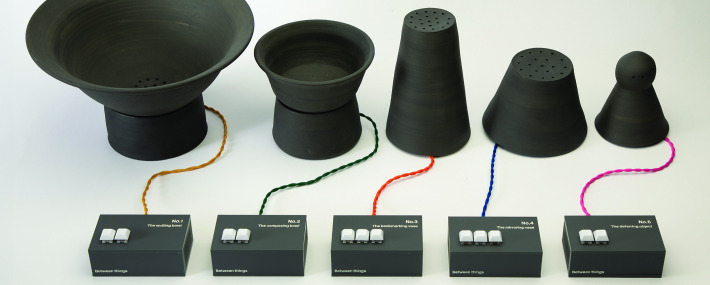
The 'Between things' objects. Alt Text: A collection of off-black bowl-shaped and conical forms. Charcoal grey acrylic boxes with white keyboard buttons are labelled from 'No.1 The waiting bowl' to 'No.5 The deferring object'. Twisted cables in ochre, green, orange, blue and pink connect each box to its ceramic object. All photography credits: Studio Ordinary.

Five objects in a collection called *Between things* embody the implications of augmented communication taking longer than, and having a different rhythm to, non-augmented communication (Figure 5). They invite reflection on how both conversational partners, not just the augmented communicator, might adapt. How might AAC be conceived as a shared resource? This is a very different starting point than framing it as a prosthesis or even an implant. In some ways, the *Between things* objects relate to speculative design (Dunne and Raby 2013). This is a methodology in which designers create objects for a speculative future, affording shared reflection on how technology might develop in the future and how people might change as it does. In a field-like AAC in which the future can feel dominated by emerging technologies—currently AI and BCIs—we might have created speculative AAC devices in order to critique this technological determinism, but the role of the *Between things* is more illustrative, to embody abstract issues rather than proposing a specific future. So, complementary to all the AI-powered development attempting to eliminate time delays in AAC altogether, the *Between things* explore what it might mean to approach uncomfortable silences and turn-taking from perspectives that are both more pragmatic and also afford more agency to an AAC user.

The first object in the collection, *Between thing No.1: the waiting bowl*, is a large ceramic bowl placed on a table between conversational partners. An embedded speaker can be activated physically, with a button, or wirelessly from an AAC device, diffusing ambient music and sounds into the room. This fills a silence that can open up in a conversation when someone is using AAC. It acknowledges the silence—unapologetically, one might say. This object aims to seed conversations about timing and turn taking with AAC. In the time it can take an AAC user to compose a response, conversational partners may start talking among themselves, or the conversation might have moved on altogether (Higginbotham and Wilkins 1999). This lack of agency to direct or hold a conversation can be frustrating and undermining.

In the past, Preece has wanted people to know that he is preparing a response to them, or about to say something else. He discussed this wish with the manufacturer of his AAC and as a result his current device now includes a secondary screen on the back, facing his conversational partner, which can display an animation of three dots to represent typing. In an analogous role, *Between thing No.2: the composing bowl* emits the sound of typing, letting a conversational partner know that a response is being prepared. But without looking at, and perhaps becoming fixated with, a screen that might distract from eye contact.

Turn-taking can be difficult to navigate, especially in conversations involving more than two people. While an AAC user is composing their contribution, the conversation might have moved on (Higginbotham, Fulcher, and Seale 2016), and so, by the time an utterance is spoken, the original context might have been lost. *Between thing No.3: the bookmarking vase* constantly records in the background (only), so that an AAC user can capture the point that they will respond to and play it back later, when they are ready to speak, reminding everyone of this context. Alternatively, *Between thing No.4: the mirroring vase,* harvests the last utterance and replays it in the AAC user’s digital voice, as a way of deliberately seizing and holding the floor.

The final object, *Between thing No. 5: the deferring object*, takes the form of an abstracted head, with two dots alluding to eyes and therefore an implied direction of gaze. When triggered, it rotates to face the AAC user, a behaviour that has a paradoxical role: the intention being to divert attention away from itself and towards the AAC user, so as to make their body language more noticeable to a conversational partner. This is a counter-intuitive approach for an AAC device and we hope that it is thought-provoking in the context of the multimodality of communication, already highlighted by others (Goodwin 2004; Higginbotham 2010; Valencia *et al*. 2021). AAC can inadvertently get in the way, yet the ways in which it might not—in which it might even harness other modalities—feel underexplored.

**The** ***Between things*** **objects are something that do something—but they’re more to fill a blank in the conversation or a blank in the idea in your mind. Like a framework, something to support or scaffold how to think around. And I think we came up with some thoughts yesterday that we wouldn't have done had that not been there. Like the‘x’in algebra. That’s how I see it. It’s a variable and you don’t know what it is, but you can’t just call it nothing because there’s more than one thing that exists.**

## Owning the future

**Recently I updated my AAC device’s software, and to my surprise my predictive text was reset and changed. I talked to the company who provides my AAC and they said that the newer version would quickly catch up, but it still hasn’t. Before, if I wrote** ‘**I love you’,** ‘**Emma’ would come up—this doesn’t happen anymore. Not only does this highlight my lack of ownership in the decision-making process around my voice, but also my sense of ownership of current and previous features. It’s unfair that without warning and without an opportunity to assert agency, elements of my voice can be taken away from me.**

The withdrawal of features that they have got used to is something that people who use AAC experience and resent. ‘Ownership’ therefore is not just in the present tense: it is also about retaining possession and use of an existing technology even after it becomes reframed as a past technology. Even at this basic level—hardly the stuff of imagining technology futures—a feeling of ownership can be frustrated.

It was not only because Stephen Hawking was its most famous user, that he could be said to have had ownership of his computer voice in a way that most people who used a similar voice did not: he also was in control of its continuity. As speech synthesis was improved, companies ceased to support the older voices on new platforms. Hawking had by then become attached to his original voice and wanted to retain it. In the unusual circumstances of being surrounded by engineers at the University of Cambridge, together with support from Intel, he commissioned a version of the obsolete voice that ran on the new technology.

A wish for self-determination is one of the things that has led to the growth of a culture of ‘hacking’ of assistive technologies (Hurst and Tobias 2011). ‘We are the original lifehackers’ says advocate and activist Liz Jackson (Jackson 2018), noting that credit is often not given for disabled people’s creativity, recasting them as recipients of design rather than designers themselves.

Widespread ownership in this sense implies not just ‘open source’, but ‘open access’: open source might just be accessible and understandable to technical experts, while open access implies being accessible to more people, to most people. The issue of continuity is all part of this: people’s communication and identity will be invested in this technology and it must not be able to be taken away from them.

Contrast this with the wide distribution of developing AI, simplistically (and misleadingly) referred to as the ‘democratisation of AI’. Most people who use current AI models have little to no control of the system, or understanding of what happens within its black box (Waller 2024). This compounds important issues of privacy that already affect people who use AAC. Given that AI typically requires vast data sources to train itself, would an AAC user be prepared to compromise their privacy in return for a potentially faster, more articulate or expressive AAC device?

As an alternative, more open technologies could afford more transparency. Enabling more expression of voice in AAC might not imply inventing a new technology as much as enabling a new combination of, or workflow between, existing technologies. If this is to be under the direction of disabled people, this could necessitate negotiating a relationship or license agreement around this. It might involve the complexity of necessary permissions that could not be subsequently taken away, such as Creative Commons Attribution (CC BY), a license that allows others to use and modify a work, as long as the original author is acknowledged and other conditions are met.

Beyond the ownership of existing technology is the question of who directs the development of the technologies that underpin AAC. Brought into the context of disability futures, this individuality leads us to a stance that qualities of voices need to be able to be created *by*, not *for* or even just *with*, disabled people; on their own terms—prioritising things, such as the individual sexuality of people who use AAC and about how AAC could do more to support this aspect of their identities, for instance (Preece 2024), that imply deep agency and accountability in social interactions.

This ownership extends to future research and development in the field as well (Pullin *et al*. 2017). Not just including AAC users in the design but writing proposals together for future research—including fundamental and speculative research. As coresearchers, not ‘subjects’ of research or even just participants, which opens up all kinds of further questions: how can people who use AAC become therapists, engineers, designers, researchers? At the moment this is unusual—are education and these professions themselves accessible and inclusive?

These are the highest level issues and they must connect to the practical details: AAC cannot be ‘customised’ at the point at which it is obtained: its use has the potential to change how the individual structures their thoughts and their communication. Therefore, their AAC needs to evolve as they do. In other words, the ability to reconfigure, make and remake one’s AAC needs to be perpetual: why it is changed and how it is changed should be under the control and ownership of the individual.

**My current AAC suits me but it is a work in progress and will never be complete.**

## Data Availability

Data sharing is not applicable as no datasets generated and/or analysed for this study.
